# Postoperative Perianal Abscess and Concomitant Anorectal Fistula: An Extremely Rare Complication After Emergency Transanal Hemorrhoidal Dearterialization With Mucopexy for Hemorrhoidal Disease

**DOI:** 10.1155/cris/1465838

**Published:** 2025-08-08

**Authors:** Charito Chatzinikolaou, Konstantinos Perivoliotis, Amalia Moula, Kyriakos Psarianos, Alexios Stavrou, Ioannis Baloyiannis

**Affiliations:** ^1^Department of Surgery, General Hospital of Volos, Polymeri 134, Volos 38222, Greece; ^2^Department of Surgery, University Hospital of Larissa, Mezourlo, Larissa 41110, Greece

## Abstract

We report the rare case of postoperative perianal abscess after emergency transanal hemorrhoidal dearterialization (THD) with mucopexy for Grade III hemorrhoidal disease (HD). A 68-year-old male presented to our hospital with rectal bleeding due to HD Grade III. He underwent THD with mucopexy with an uneventful postoperative recovery. The patient was evaluated on the 15^th^ postoperative day due to perianal pain without any abnormal laboratory and imaging findings. One month postoperatively he presented with perianal edema and pus discharge. During the rectal examination, a perianal abscess with a concomitant fistula was identified and was confirmed with an MRI scan. He was submitted to abscess drainage and seton placement. This report aims to raise awareness among colorectal surgeons about the risk for this specific complication during the postoperative period. Further studies, are needed so that the etiopathology of this condition is identified and the risk factors can be controlled and avoided.

## 1. Introduction

Hemorrhoidal disease (HD) is a benign anorectal disorder that affects an important percentage of the population worldwide and has a notable impact on their quality of life [1]. It is estimated that at least 20% of the population will be diagnosed with hemorrhoids throughout their life and possibly treated surgically [1, 2].

There are several methods regarding the treatment of HD based on the grade, the severity of symptoms and patient preferences. Transanal hemorrhoid dearterialization (THD) is one of them and offers an effective treatment avoiding the unfavorable outcomes of an excisional approach, including increased morbidity, postoperative pain, and decreased quality of life [3, 4]. This technique is based on the ligation of the hemorrhoidal arterial flow to the hemorrhoidal plexus through a doppler-guided transducer involved in the anoscope. As a result, the arterial overflow of the hemorrhoidal piles is reduced and the symptoms are resolved with the preservation of the hemorrhoidal tissue. Usually, it is accompanied by mucopexy where the prolapsing tissue is repositioned to the anatomical site [3, 5, 6].

THD is a generally safe procedure with minor complications. The most reported complication is tenesmus which can result in rectal discomfort and pain [6]. Other described complications are urinary retention, bleeding, hemorrhoidal thrombosis, acute anal fissure, pain, pruritus and transient submucosal hematoma [3–6].

Postoperative abscess is a notably rare complication after THD. In a prospective study by Bjelavonic et al. [7], a 0.8% rate of abscess formation after THD was reported. Moreover, the potential risk factors for this rare complication have not been adequately clarified [7]. Additionally, to the best of our knowledge, there is only one published study that described the postoperative formation of a horseshoe perianal abscess after THD [8]. Subsequently, in this report we describe the rare case of a patient who developed a postoperative perianal abscess and a concomitant anorectal fistula a month after emergency THD for Grade III hemorrhoids.

## 2. Case Presentation

A 68-year-old male presented to our hospital due to anal bleeding. He had a medical history of coronary disease with bypass surgery 10 years ago and a percutaneous coronary intervention 4 years ago for which he received clopidogrel. During his hospital stay he was transfused with 5 units of red blood without laboratory stabilization. The endoscopic examination was normal, and he was diagnosed with hemorrhoid disease stage III. Endoscopy did not reveal any inflammation in the bowel and rectal mucosa. As a result, he underwent an emergent THD procedure with mucopexy. The procedure was performed under spinal anesthesia in the lithotomy position. Perioperative antibiotic prophylaxis included the administration of cefoxitin and metronidazole. Initially, anoscopy confirmed the absence of any perianal abscess or fistulae. A doppler ultrasound probe was used, and the six terminal branches of the superior rectal artery were identified and ligated with a figure of “8” vicryl stitch which was completed with a mucopexy. The patient returned to the department and experienced urinary retention which was treated with transient urinary catheterization. He was discharged 2 days postoperatively with no need for transfusion.

**Figure 1:**
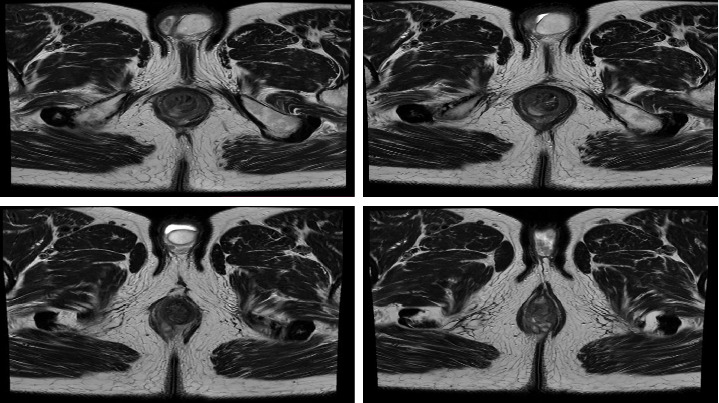
The abscess and concomitant fistula as shown at 7 o'clock in consecutive axial T2sFOV series.

The patient presented with anorectal pain and mucous discharge 15 days postoperatively. Physical examination did not reveal any dubious findings. He underwent a CT scan without any pathological signs and with normal laboratory tests. Symptomatology was resolved after 1 day of hospitalization and the patient was discharged with prescribed analgesics and laxatives. However, the patient referred to our hospital again 1 month postoperatively with edema and pus discharge from the perianal area. During the physical examination an abscess with a concomitant perianal fistula was identified. MRI scan (Figure 1) confirmed these findings. From the laboratory results, the white blood cell count was 6.4 ×10^9^/L (normal 4.6–10.2 ×10^9^/L), while a hemoglobin level of 11.9 g/dL (normal 13.5–17 g/dL) and CRP level of 2.75 mg/L (normal 0–5 mg/L) was found. As a result, the patient underwent perianal abscess drainage and seton placement in the fistula tract. The patient was relieved from the symptoms, tolerated a regular diet and was discharged on the first postoperative day without any complications.

## 3. Discussion

HD is among the most common benign anorectal diseases and can severely affect quality of life and social wellbeing [7]. According to current literature, the prevalence of HD is 4.4%, with a peak in the 45–65 years old range and a decline after the age of 65 [9]. Furthermore, the intensity of presenting symptoms is negatively correlated with the health-related quality of life, physical and mental health indexes, which improve after the successful treatment of the disease [10].

Optimal management of hemorrhoids encompasses a wide range of conservative measures, interventional techniques and surgical operations [11–13]. Current guidelines suggest that the initial approach should include dietary and lifestyle modifications, alongside the application of local medications [11–13]. In case of conservative treatment failure or symptom persistence, surgical management should be considered [11–13].

Colorectal surgeons have at their disposal multiple techniques for the management of hemorrhoids, with significant differentiations in terms of pathophysiological approach, invasiveness, postoperative outcomes and recurrence risks [14–16]. Overall, excisional techniques are recommended for higher grade disease, with the disadvantage of postoperative pain and prolonged hospitalization [11–13].

THD procedure was proposed by Morinaga et al. [17] in 1995 as a minimally invasive alternative for the treatment of HD [18]. Its principle is the ligation of the terminal branches of the superior rectal artery selectively with the aid of a doppler-guided ultrasound probe [7]. The proctoscope has a specific pivot hole that prevents from performing deep stitches and thus they are calibrated to the depth that is needed so that the artery is safely ligated that is 6 mm maximum. This is an advantage of this method where only mucosa and submucosa are involved, and the rectal wall is not perforated. In case of prolapse, mucopexy is also performed with the removal of the upper part of the proctoscope and the formation of continuous sutures on the mucosa and submucosa with steps of 0.5 cm below the dearterialization point [3].

Moreover, THD is associated with a safe and efficient profile [14–16]. This method results in fewer postoperative complications and an acceptable symptom relief rate. More specifically, THD is related to significantly lower postoperative pain compared to the excisional procedures and is highly recommended for Grade II and III HD [5].

Overall, perianal abscess after HD surgery is a rare complication [19]. In previous reports, the rate of perianal fistula and abscess following hemorrhoidectomy is estimated to be between 0.5% and 4% [19]. Indeed, in a 10-year retrospective study by Moldovan et al. [20], the pooled surgical site infection rate after hemorrhoidal surgery was 0.6%.

In terms of THD, due to the scarcity of these adverse events, there is limited bibliography assessing this complication. Bjelavonic et al. [7] reported in his case series that the risk of abscess after THD was 0.8%. Additionally, Nakao et al. [8] described the formation of a horseshoe abscess after the procedure, which was, also, treated operatively [8].

In our case the patient was submitted to an emergency operation due to persistent symptomatic HD that required multiple transfusions. Baseline evaluation with endoscopy did not reveal any intraluminal disease and no other risk factors for surgical site infections were identified. Moreover, intraoperative anoscopy did not show any dormant fistula tract that could be activated postoperatively. Although, a direct pathophysiologic pathway cannot be confirmed, the emergency status, the disease stage, the level of prolapse, and the need for operative treatment during a hemorrhoidal crisis could be potential contributing factors.

As a result of these scarce reports, valid data regarding the true incidence and the associated risk factors of this complication are still lacking. Thus, clinicians should have increased awareness for the risk of abscess formation during a symptomatic postoperative period and further research regarding the potentially modifiable risk factors should be considered.

## 4. Conclusions

THD is a safe and effective treatment for HD. In this report, we described the rare case of a postoperative perianal abscess with a concomitant fistula in a patient submitted to emergency THD for Grade III hemorrhoids. Despite its rarity, colorectal surgeons should be alert for the potential of this complication. Further clinical trials are required to assess and identify any modifiable risk factors.

## Data Availability

The data that support the findings of this study are available from the corresponding author upon reasonable request.
